# Differential Tissue-Specific Jasmonic Acid, Salicylic Acid, and Abscisic Acid Dynamics in Sweet Cherry Development and Their Implications in Fruit-Microbe Interactions

**DOI:** 10.3389/fpls.2021.640601

**Published:** 2021-02-02

**Authors:** David H. Fresno, Sergi Munné-Bosch

**Affiliations:** ^1^Department of Evolutionary Biology, Ecology and Environmental Sciences, University of Barcelona, Barcelona, Spain; ^2^Institute of Nutrition and Food Safety (INSA), University of Barcelona, Barcelona, Spain

**Keywords:** exocarp, jasmonic acid, microbiome, plant-microbe interaction, salicylic acid, *Prunus avium* L.

## Abstract

Sweet cherry is an important non-climacteric fruit with a high commercial interest, but exploitation of sweet cherry trees (*Prunus avium* L.) in orchards is usually subject to important economic losses due to fruit decay by pathogenic fungi and other microorganisms. Sweet cherries development and ripening are characterized by profound physiological changes in the fruit, among which the phytohormone abscisic acid (ABA) plays a pivotal role. In addition, sweet cherries are usually affected by fruit decay pathogens, and the role of other stress-related hormones such as jasmonic acid (JA) and salicylic acid (SA) may also be of paramount importance, not only from a developmental point of view, but also from a fruit-microbe interaction perspective. Here, a tissue-specific hormone quantification by LC-MS/MS, including the contents of JA, SA, and ABA, in the fruit exocarp and mesocarp of sweet cherries during fruit development from trees growing in a commercial orchard was carried out. Additionally, this study was complemented with the characterization of the culturable epiphytic and endophytic microbial communities of sweet cherries at various stages of fruit development and during cracking lesion formation. Our results revealed a completely differential behavior of phytohormones between both tissues (the exocarp and mesocarp), with a more dynamic exocarp in front of a more stable mesocarp, and with marked variations during fruit development. Microbial epiphytic community was mainly composed by yeasts, although rot-causing fungi like *Alternaria* spp. were always also present throughout fruit development. Endophytic colonization was poor, but it increased throughout fruit development. Furthermore, when the exocarp was naturally disrupted in sweet cherries suffering from cracking, the colonization by *Alternaria* spp. markedly increased. Altogether, results suggest that the fruit exocarp and mesocarp are very dynamic tissues in which endogenous phytohormones not only modulate fruit development and ripening but also fruit-microbe interactions.

## Introduction

Sweet cherry (*Prunus avium* L.) is an important stone fruit crop which has recently received increased interest due to both its organoleptic and visual characteristics and high nutritional value (Kelley et al., 2018). Most sweet cherries are marketed globally as a fresh product (Chockchaisawasdee et al., 2016) but they are highly perishable due to different factors, among which microbial decay caused by pre‐ and post-harvest pathogens is one of the most relevant (Alonso and Alique, 2006). Although necrotrophic filamentous fungi like *Botrytis cinerea* and *Alternaria* spp. are known to be the main pathogens affecting cherry fruits (Habib et al., 2015; Chockchaisawasdee et al., 2016), very little is still known on how endogenous phytohormone contents might influence their decay. Sweet cherry is a non-climacteric fruit, which is characterized, in contrast to climacteric fruits, by a progressive abscisic acid (ABA) accumulation, a lower respiration rate and almost no ethylene release during ripening (Pérez-Llorca et al., 2019). However, although cherry fruits are composed by an outer layer, the exocarp, an edible mesocarp, and a stony endocarp, no studies have focused thus far on better understanding the contribution of these tissues on fruit decay.

Plant-microbe interactions and their underlying mechanisms are increasingly receiving attention in the exploitation of commercial orchards of stone fruity trees because of their economic relevance. In this respect, not only plant pathogens, which are able to colonize the plant inner tissues causing disease to the host (Schlechter et al., 2019), but also epiphytic microorganisms, those that live on the plant surfaces, and endophytes, when they colonize inner tissues without affecting the host’s fitness (Takken and Rep, 2010), may play a major role. Furthermore, plant physical barriers, like the cuticle or cell walls in leaves or the exocarp in fruits, as well as the immune responses elicited by endogenous phytohormones, keep microbial communities under control (Schlechter et al., 2019). Indeed, physical barriers and immunity are tightly interconnected, so that when physical barriers fail, immune responses such as pathogen associated molecular pattern (PAMP)‐ and effector-triggered immunity (PTI and ETI, respectively) take place to halt the microbial invasion (Bigeard et al., 2015; Cui et al., 2015).

Plant immune responses have been extensively studied, and the role of specific phytohormones like salicylic acid (SA), jasmonic acid (JA), ABA, or ethylene has been described in detail in previous studies using model plants (reviewed by Pieterse et al., 2012). In addition, the role of these hormones in physiological processes like ripening of non-climacteric fruits is already well-known (Leng et al., 2014; Tijero et al., 2019). However, it remains unclear how the interactions between these hormones and ripening may affect fruit specific immune responses to pathogens and, eventually, fruit susceptibility to fruit decay, most particularly in fruits of non-model plants. Previous research proved that ABA has a negative effect in tomato resistance to the necrotrophic pathogen *B. cinerea*, and ABA signaling has been shown to antagonize with SA and JA-dependent responses to biotic stress in tomato plants (Asselbergh et al., 2007; Pieterse et al., 2012). However, due to technical limitations and the difficulty of performing studies in commercial orchards, most of this information has been obtained in model plants and using whole fruits. However, plant immune responses take place in a localized manner, and the role of different plant tissues in these responses may be relevant in plant-pathogen interactions. Thus, it is highly relevant to monitor how hormones vary during fruit development at the tissular level to unravel specific responses in the exocarp and mesocarp of fruits.

It is generally known that fruits become more susceptible to pathogen attack at later stages of ripening, although the main molecular and biochemical mechanisms underlying this susceptibility have not been unveiled yet (Forlani et al., 2019). Besides, one of the main physical barriers that protects fruits from pathogens is the exocarp. Fruit cracking is a physiological process in which lesions are produced on the fruit skin and flesh, disrupting the fruit’s main physical barrier, leading to severe microbial infections of the fruit (Correia et al., 2018). Thus, finding new ways to protect fruits, and especially sweet cherries from microbial decay is of major importance for fruit production. Previous studies have described the role of different phytohormones during sweet cherry fruit development, ripening, and overripening (Teribia et al., 2016; Tijero et al., 2016), and others have described the microbiome of different fruits (Granado et al., 2008; Zhang et al., 2018). In the present study, we combine these approaches by studying sweet cherries from a commercial orchard on the tree with the aim of unveiling the possible underlying physiological and biochemical causes of fruit decay in sweet cherries during ripening. With this aim, we examined the variations in the endogenous contents of the hormones ABA, SA, and JA at a tissue-specific level (exocarp and mesocarp) throughout fruit development, combined with a microbial analysis of the culturable epiphytic and endophytic populations. As profound physiological changes take place during fruit development, we hypothesized that variations in ABA, SA, and JA contents in different fruit tissues occur, which might have implications on the fruit culturable microbiome. We found that both the exocarp as a physical barrier and the hormone dynamics in specific fruit tissues may influence fruit responses to fruit microbiome and pathogens.

## Materials and Methods

### Experimental Design, Treatments, and Samplings

Sweet cherries (*P. avium* L. var. Prime Giant; rootstock SL-64) were obtained from eight trees (hereafter referred as *n* = 8) located in a commercial sweet cherry orchard at Partida Vall del Sector III (Lleida, Spain). Trees were organized in rows (5 m separation between rows) and all the fruit samples were collected from trees at the same row (with a separation of 3 m between trees). Fruits were harvested at stages of development I, II, and III and at commercial harvest on May 10th, 14th, 21st, and 31st, 2019, respectively, between 9 and 10 am local time. Fruits were visually characterized, and stages defined as described by Teribia et al. (2016) but with some modifications: stage I, green fruits; stage II, fruits with green coloration turning to yellow and red; stage III, red-pink color with maximum 30% green; and Harvest, commercial harvest. Fruits were always collected from bunches located in the outer branches of each tree.

For fruit hormone analysis, a pooled sample of six fruits per tree was used as one replicate and a total of eight replicates from different trees were used. Every cherry was quickly cut by half to discard the endocarp, immediately frozen in liquid nitrogen, and stored at −80°C until further processing. For microbial analysis, a pooled sample of three fruits per tree was used as one replicate and a total of eight replicates from different trees were used. Cherries were introduced inside sterile bags, and immediately transported under refrigeration to the lab for further processing.

Finally, for cracking analyses, three sweet cherries suffering from natural cracking were collected at harvest from another tree located at the same row, stored in sterile bags, and transported under refrigeration to the lab until further use.

### Microbial Extraction and Isolation

Under sterile conditions, three cherries were gently shaken for 5 min inside a sterile bottle containing a saline solution (NaCl, 9 g/L) to extract the fruit epiphytic microorganisms. Afterward, 10 ml of that solution was collected and stored in sterile falcon tubes at 4°C until further processing. After epiphyte extraction, three measures from each cherry (Supplementary Figure 1) were taken to estimate the skin surface (cm^2^). Cherries were surface sterilized by immersion in 70% ethanol for 5 min, in 1% sodium hypochlorite for 2 min, and rinsing three times with sterile distilled water. For each sample, 10 ml of the last rinsing water was collected and stored as a sterility control.

For endophyte extraction, after surface sterilization, one half from each cherry was cut and weighed under sterile conditions. Then, they were ground on a sterile mortar containing 10 ml of saline solution using a sterile pestle, and the homogenate was collected and stored in sterile falcon tubes at 4°C until further use. Prior to grinding, both mortar and pestle were washed with 10 ml of sterile distilled water, which were stored and further used as a sterility control.

The falcon tubes containing epiphytes and endophytes, as well as the sterility control tubes, were centrifuged for 15 min at 6000 rpm (Hettich Universal 32R) and the microbial pellet was resuspended in 3 ml of fresh sterile saline solution. For the epiphyte suspension, a 1:10 dilution was done, and 500 μl of it were plated on nutrient agar (NA) and potato dextrose agar (PDA) plates. For the homogenate containing endophytes and the sterility controls, 500 μl of the suspension were directly plated on NA and PDA plates. The plates were incubated at room temperature (22 ± 1°C) and dark conditions for 7 days, when colony forming units (CFU) were scored. Eventually, epiphytic CFU per cm^2^ of fruit exocarp, per fruit, or endophytic CFU per gram of fresh mesocarp were calculated. Microbial isolates were tagged as yeast, bacteria, or other fungi. The proportion of every microbial group in each sample was calculated. The most abundant yeast and bacterial isolates were morphologically characterized according to their colony type, colony edge, colony color, and texture (Krishnan et al., 2012). The most frequent fungal isolates were morphologically identified by colony structure and microscopic characteristics.

### Tissue-Specific Hormone Analysis

Exocarp and mesocarp from each cherry were separated for a differential hormone analysis of both tissues. The exocarp of every frozen cherry (−80°C) was separated from the mesocarp using a scalpel by scraping the fruit surface. Afterward, both tissues were ground separately using a pestle and a TissueLyser (Qiagen). These processes were always performed using liquid nitrogen to avoid sample defrosting. Plant hormones, including ABA, SA, and JA, were extracted and quantified by LC-MS/MS. Ground exocarp (50 mg) and mesocarp (100 mg) samples were extracted with 250 μl pure methanol and deuterium-labeled internal standards (d6-ABA, d5-JA, and d4-SA) before ultrasonication and vortexing (Branson 2510 ultrasonic cleaner, Bransonic, United States) for 30 min. After centrifugation, the supernatant was collected and the pellet was re-extracted using the same procedure. Both supernatants were merged and filtered through a 0.22 μm PTFE filter (Waters, United States). Hormone levels were analyzed by UHPLC-ESI-MS/MS as described by Müller and Munné-Bosch (2011). The ultrahigh-performance liquid chromatography (Aquity UPLCTM System, Waters Milford, MA, United States) was coupled to a triple quadrupole mass spectrometer (API 3000, PE Sciex, Concord, Ont., Canada). A Kinetex C18 (Phenomenex Inc., United States) column (1,7 μm, 100 × 2.1 mm) was used. Solvent A was water with 0.05% glacial acetic acid and solvent B was acetonitrile with 0.05% glacial acetic acid. Flow rate was set at 0.6 ml/min. Quantification was made considering recovery rates for each sample by using the deuterium-labeled internal standards as described by Müller and Munné-Bosch (2011).

### Fruit Cracking Analysis

Three cherries suffering from natural cracking were collected at harvest. Every cherry had the same degree of cracking located on the fruit cheek. Each cherry was divided into three different regions, including the cracking lesion itself, the area around the cracking lesion (zone 1), and the region opposite to the cracking lesion (zone 2). For the isolation of endophytic microorganisms from cracked cherries, fruits were surface sterilized by immersion in 70% ethanol for 5 min, in 1% sodium hypochlorite for 2 min, and rinsed three times with sterile distilled water. Five-hundred microliter of the last rinsing water was plated on PDA plates to certify complete surface disinfection. For each cherry, using a sterile scalpel, approximately 25 mesocarp pieces (0.5 × 0.5 cm) from zone 1 and zone 2 were cut and plated on PDA plates. Plates were incubated at room temperature (22°C) and dark conditions for 7 days, and microbial outgrowth from the pieces was scored. Microbial isolates were morphologically characterized (Krishnan et al., 2012) and, if possible, identified. For *Alternaria* spp. colonization percentage calculation, for each zone, the number of pieces showing pathogen outgrowth was divided by the total number of mesocarp pieces plated.

### Antagonistic Activity of Endophytic Isolates Against *Botrytis cinerea*

The “killer stroke” method was performed as described before (Pretscher et al., 2018). In brief, microbial isolates showing an endophytic behavior in healthy or cracked cherries were streaked in the centre of PDA plates and incubated for 4 days at 22°C. *Botrytis cinerea* plugs from a well-grown PDA plate were placed 2–3 cm away on both sides of the streak. Plates were incubated at 22°C for 7 days, and fungal growth was monitored daily to observe *B. cinerea* growth inhibition.

### Statistical Analysis

A linear regression model was performed to predict the cherry skin surface out from three specific measures (Supplementary Figure 1): (a) fruit width, (b) fruit height from the apex to the base of the peduncle, and (c) width at the top of the fruit. After measuring (a), (b), and (c) on 24 cherries at stages III and harvest, the skin from these fruits was scanned and its area (cm^2^) was quantified using ImageJ. A linear regression model was performed, showing the following model to predict fruit skin area: area (cm^2^) = −33.6695 – 0.3837(b) + 20.064(c). The (a) variable was discarded during the analysis due to multicollinearity.

To estimate the effect of “Stage of Development” in epiphytic populations and epiphytic composition, mean values were tested by one-way ANOVA and *post-hoc* Tukey honestly significant difference (HSD) test to determine statistical differences. To determine the effect of “Stage of development” in endophytic colonization, a non-parametric Kruskal Wallis test was performed, followed by a *post-hoc* Fisher least significant difference test. To analyze differences in *Alternaria* spp. colonization rate between zones in cracked cherries, a non-parametric Kruskal Wallis test was performed. Finally, to determine the effect of “Stage of development” and “Fruit tissue” in endogenous ABA, SA, and JA contents, mean values were tested by two-way ANOVA followed by *post-hoc* Tuckey HSD. In addition, Spearman’s rank correlation analyses were performed between hormone levels at different tissues and endophyte CFU/gFW. For ANOVA analyses, data were log_10_ or square root transformed to accomplish normal distribution and homogeneity of variance, if needed. In all cases, differences were considered significant at a probability level *p* < 0.05. All statistical tests were performed with RStudio.

## Results

### Tissue-Specific Hormone Contents Vary Depending on the Stage of Development

**Figure 1:**
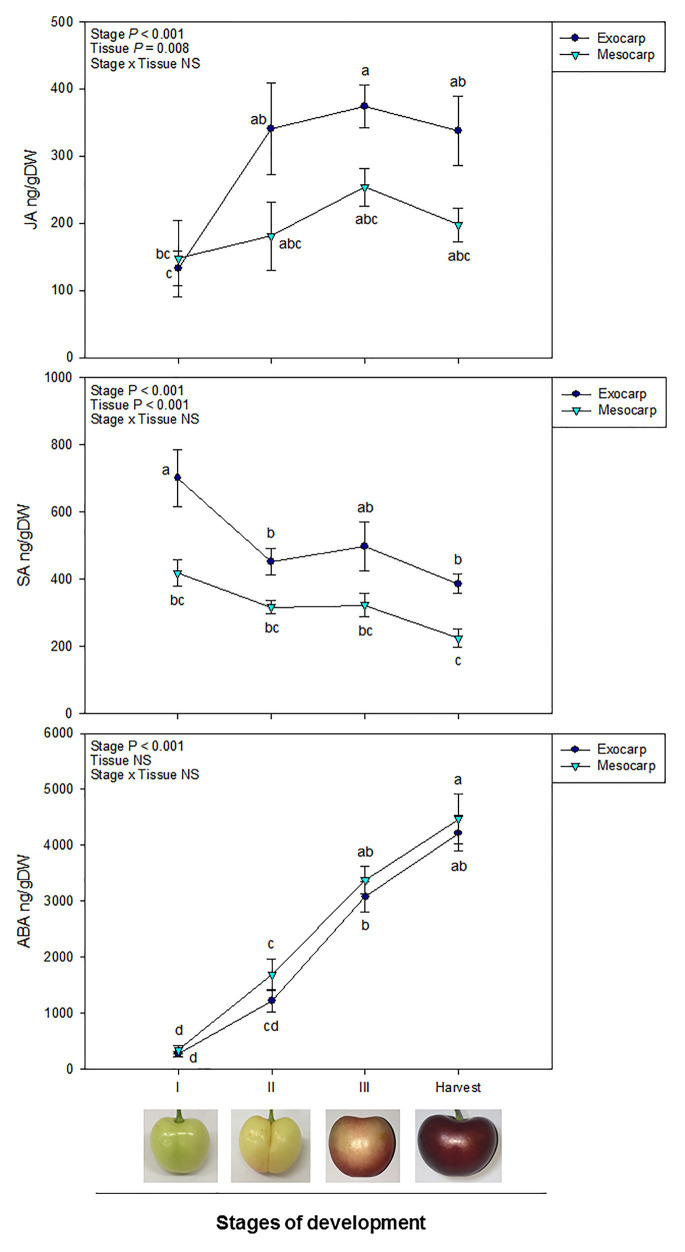
Endogenous contents of jasmonic acid (JA), salicylic acid (SA), and abscisic acid (ABA) in the exocarp and mesocarp of sweet cherries during different stages of natural fruit development in trees from a commercial orchard. Data are means ± SE of *n* = 8. Statistical comparisons were performed by two-way ANOVA, followed by a *post-hoc* Tukey honestly significant difference (HSD) test. Different letters indicate significant differences between stages of development and tissues (*p* < 0.05).

Endogenous contents of JA, SA, and ABA differed between the exocarp and mesocarp at different stages of development (Figure 1). A clear differential behavior in both JA and SA contents was observed in the exocarp in comparison to the mesocarp. A significant effect of the “Stage of development” and “Tissue” was observed in the endogenous contents of both JA and SA, although the interaction of both factors was not significant.

In the case of JA, an increase in JA contents was observed at initial steps of fruit development in the exocarp, specially between stages I and II, contents remaining stable afterward until harvest. On the contrary, in the mesocarp, JA contents did not significantly vary all through fruit development (Figure 1).

Regarding SA, there was a significant drop in the SA content in the exocarp at the beginning of fruit development, from stage I to II. Afterward, SA content remained relatively constant until harvest. No differences in SA levels, however, were reported in the mesocarp, where endogenous SA barely decreased throughout development. Furthermore, SA differentially accumulated in the exocarp, showing higher levels than in the mesocarp both at the beginning of fruit development (stage I) and at harvest (Figure 1).

In contrast to JA and SA contents, ABA levels gradually increased during fruit development regardless the fruit tissue, showing a similar accumulation both in the exocarp than in the mesocarp during fruit development and ripening (Figure 1).

Spearman’s rank correlation analyses revealed interactions between different hormone levels. A strong significant positive correlation was observed between ABA levels in the exocarp and in the mesocarp. In addition, ABA levels in the exocarp were also correlated with other hormones, like JA (positively) and SA (negatively), not only in the exocarp but also, although to a lesser extent, in the mesocarp (Table 1).

**Table 1:** Spearman correlation analysis between exocarp and mesocarp hormone levels and endophytic colony forming units (CFU) per gram of fresh weight.

	ABA-meso	SA-exo	SA-meso	JA-exo	JA-meso	CFU/gFW
ABA-exo	0.848^***^	−0.390^*^	−0.482^**^	0.566^***^	0.386^*^	0.430^*^
ABA-meso		−0.393^*^	−0.534^**^	0.456^**^	0.337	0.481^**^
SA-exo			0.231	−0.266	−0.227	−0.348
SA-meso				−0.198	−0.163	−0.271
JA-exo					0.624^***^	0.234
JA-meso						0.174

Rho values are represented, and significant correlations are marked by asterisks (^*^represents *p* < 0.05; ^**^represents *p* < 0.01; and ^***^represents *p* < 0.001).

ABA, abscisic acid; SA, salicylic acid; JA, jasmonic acid; CFU/gFW, endophytic colony forming units per gram of mesocarp fresh weight.

### Culturable Epiphytic Community Remained Stable Through Fruit Development

**Figure 2:**
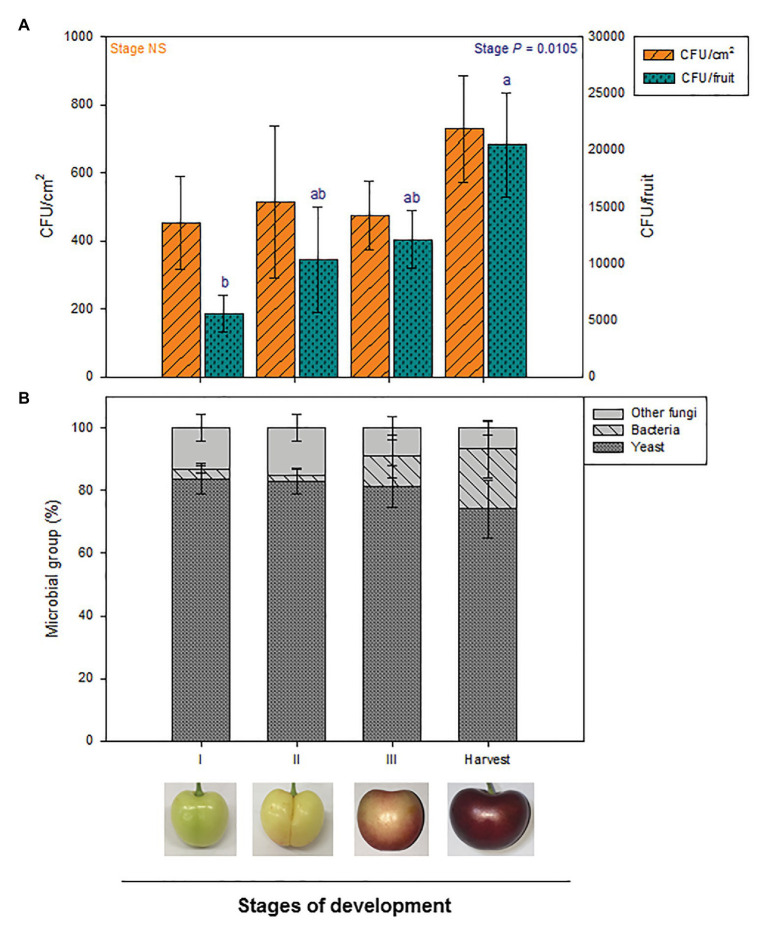
Quantification and composition of total culturable epiphytic microorganisms at different stages of fruit development in the exocarp of sweet cherries during different stages of natural fruit development in trees from a commercial orchard. **(A)** Total epiphytic CFU per cm^2^ of sweet cherry skin and per fruit are represented. **(B)** Main microbial groups present on the culturable epiphytic community of sweet cherries during fruit development. Data are means ± SE of *n* = 8. Statistical comparisons were performed by two-way ANOVA, followed by a *post-hoc* Tukey HSD test. Different letters show significant differences between groups (*p* < 0.05).

Microbial load per cm^2^ of cherry skin remained stable throughout fruit development, from the beginning of development until commercial harvest time. However, the total amount of epiphytic microorganisms per fruit significantly increased during fruit development (Figure 2A).

To have a deeper insight into the composition of culturable epiphytic microorganisms on sweet cherry surfaces, epiphytes were categorized into three different microbial groups: yeasts, bacteria, and other fungi. Stage of development did not significantly affect proportion of microbial groups. Despite being some variability between samples, yeast was clearly the most abundant microbial group during fruit development, as 70–80% of total epiphytes belonged to this group. Other fungi and bacteria proportions varied from 7 to 16 and 0 to 20%, respectively (Figure 2B).

### Presence of Microbial Isolates, Including Fruit Rot-Causing Fungi, on the Fruit Surface

**Figure 3:**
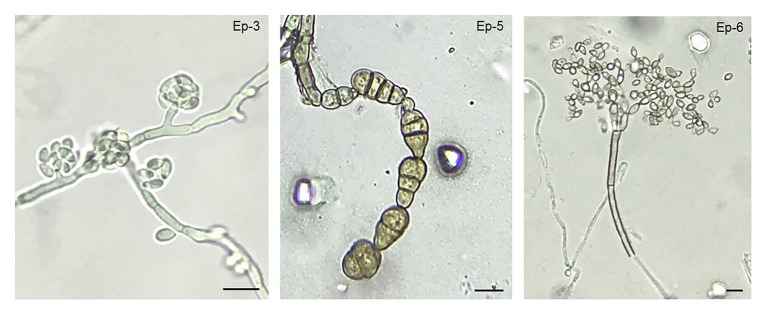
*Aureobasidium*, *Cladosporium*, and *Alternaria* as the most frequent culturable filamentous fungi found in the exocarp of sweet cherries. Micrographs of reproductive structures (conidia and conidiophores) for each main fungal epiphytic isolate are shown. According to morphological criteria, fungal isolate Ep-3 was identified as *Aureobasidium* sp., Ep-5 as *Alternaria* sp., and Ep-6 as *Cladosporium* sp. Scale bar = 10 μm.

Different yeast, bacterial, and other fungi isolates were observed during cherry development. Those microbial isolates that were found in a recurrent manner, at several stages of development, were selected, morphologically characterized and, if possible, identified according to morphological criteria. Two yeast isolates (Ep-1 and Ep-2) and one bacterial isolate (Ep-4) were repeatedly obtained from fruit surface throughout development (Supplementary Table 1). Other yeast and bacterial isolates were found as well at specific points of the experiment, but, as they were occasional observations, they were not quantified. Identification by morphological characteristics could not be carried out. In the case of other fungi, several isolates were obtained. A thorough analysis of colony morphology and microscopical characteristics revealed that *Aureobasidium*, and the rot-causing fungi *Alternaria* and *Cladosporium* were the main fungal genera present on sweet cherry skin (Figure 3). *Aureobasidium* spp. isolates presented hyphae with multiple lateral pegs, lateral accumulation of conidia and intercalary conidiophores (Zalar et al., 2008). *Alternaria* spp. isolates had characteristic catenulate and muriform conidia (Woudenberg et al., 2013) and, finally, *Cladosporium* spp. isolates showed fragile conidiophores with a really typical structure (Pitt and Hocking, 1985). Within each fungal genus, different isolates showed phenotypical variability, both in colony morphologies and in microscopic features. Representative microscopic structures from each genus are shown (Figure 3). Quantitatively, *Cladosporium* was the most abundant genus, followed by *Aureobasidium* and, finally, by *Alternaria* (data not shown).

### Fruit Colonization by Endophytes Increases During Ripening

**Figure 4:**
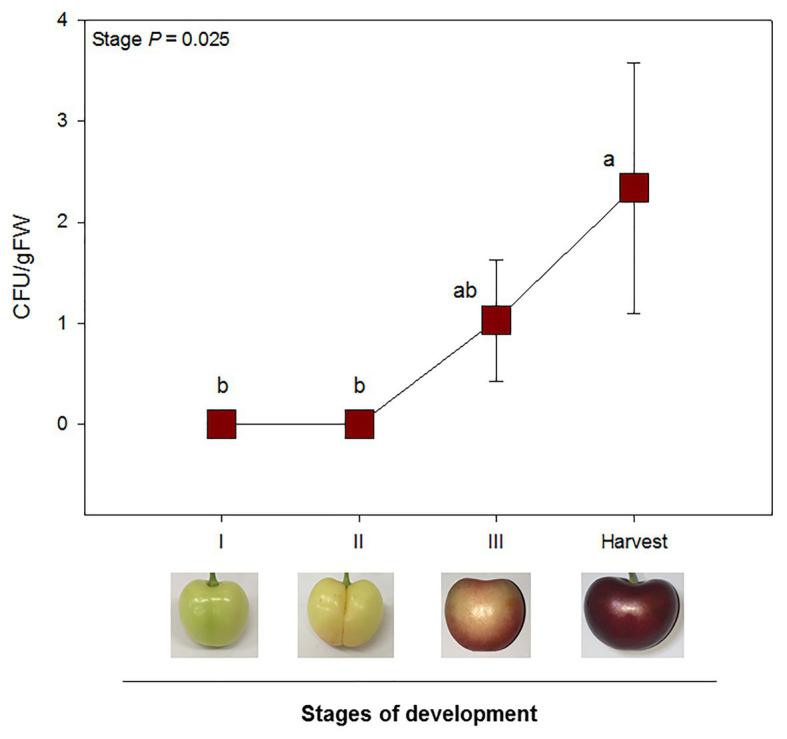
Endophytic colonization was only observed after the onset of ripening (stage III and harvest). CFU per gram of fruit flesh from sweet cherries at different stages of development are represented. Data are means ± SE of *n* = 8. A non-parametric Kruskal Wallis test was performed, followed by a *post-hoc* Fisher least significant difference test (*p* < 0.05). Different letters mean significant differences between groups.

No endophytic growth was detected at stages I or II. Mesocarp colonization was only observed after the onset of fruit ripening: at stages III and harvest. A significant increase in endophytic colonization was reported from stages II until harvest. Endophytic colonization, when present, was, however, relatively low, with mean values ranging from 1 up to 4 CFU/gFW (Figure 4). Correlation analyses between tissue-specific hormone levels and endophytic colonization revealed a significant positive correlation between both ABA exocarp and mesocarp levels and CFU/gFW (Table 1).

Three endophytic isolates were detected in the analyzed cherries, but only one of them, named as En-1, was repeatedly obtained from most of the samples at both stages III and harvest. Consequently, only En-1 was morphologically characterized (Supplementary Table 1). In addition, the antagonistic activity *in vitro* of En-1 against *B. cinerea*, a well-known rot-causing fungus, was assessed by the “killer stroke” method (Pretscher et al., 2018), showing no biocontrol ability.

### Fruit Cracking Allows Fruit Mesocarp Colonization by Epiphytic and Pathogenic Microorganisms

**Figure 5:**
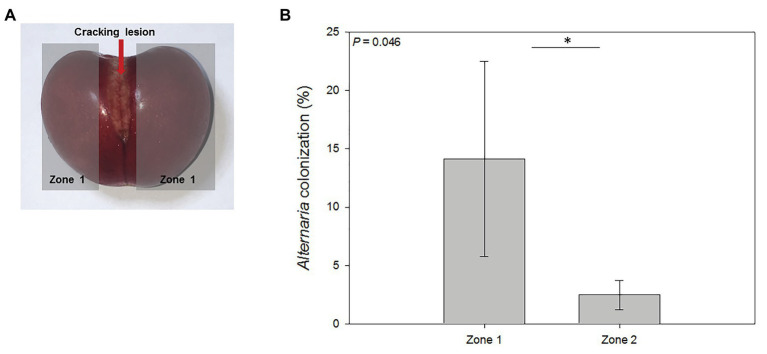
Mesocarp colonization of cracked cherries by *Alternaria* spp. **(A)** Cherries suffering from natural cracking were divided into three areas: cracking lesion, areas around the lesion (zone 1), and the area opposite to the cracking lesion (zone 2, not shown in this plane). **(B)** Percentage of endophytic colonization by *Alternaria* sp. near and opposite (zones 1 and 2, respectively) the cracking lesion. Data are means ± SE of *n* = 3. Statistical comparisons were performed by a non-parametric Kruskal Wallis test and significant differences are shown by an asterisk (*p* < 0.05).

The effect of natural disruption of sweet cherry exocarp in fruits affected by cracking on fruit-endophyte interactions was assessed. Yeast and other fungi outgrowth were observed at zone 1 (near the cracking lesion; Figure 5A). Two endophytic yeast isolates were detected and morphologically characterized (Supplementary Table 1). One of them was tagged as En-2, and the other one displayed equal characteristics to the epiphytic yeast isolate Ep-2 isolated during fruit development, and it was considered the same isolate.

Two fungal isolates were identified. On the one hand, an isolate from the genus *Aureobasidium* was obtained from one replicate in both zones of the fruit. On the other hand, *Alternaria* spp. isolates were found in every cracked fruit, colonizing both zones 1 and 2. Quantification of *Alternaria* colonization percentage of cracked cherries per zone was done, and a statistical analysis revealed that zone 2 was significantly less colonized by *Alternaria* than zones nearby the cracking lesion (Figure 5B), suggesting that the cracking lesion was the entry point for this rot-causing pathogen.

Endophytic isolates En-2, Ep-2, and *Aureobasidium* showed *in vitro* biocontrol ability of *B. cinerea* by the “killer stroke” method (Pretscher et al., 2018). *Alternaria* spp. isolates were not tested, as they are well known post-harvest pathogens.

## Discussion

A fruit tissue-specific behavior of two of the major phytohormones related with plant-microbe interactions, JA and SA, was observed in cherry fruits during fruit development in a commercial tree orchard. Previous studies on sweet cherry development have shown marked variations in different hormone levels during fruit development, showing a substantial increase of ABA, and an important decrease in JA and SA contents in whole fruits (Teribia et al., 2016; Tijero et al., 2016, 2019). However, these previous studies did not focus on the role that specific fruit tissues may have in fruit development, despite, as shown here, the differentiation between tissues may provide important hints toward better understanding the physiology and biochemistry of fruit ripening. We provide here evidence of variations in the endogenous contents of JA, SA, and ABA in the exocarp and mesocarp of sweet cherries and examine its possible relationship with the interaction of the exocarp and mesocarp of these fruits with its own microbiome and potential rot-causing pathogens. These results have important implications for biotic stress management in sweet cherry commercial orchards, as biotic stress can cause severe reductions in sweet cherry yields both during pre‐ and post-harvest.

Results showed sharp increases in JA levels in the exocarp during early stages of fruit development. More specifically, JA contents increased by 3-fold between stages I and II to remain constant later in development, while JA contents in the mesocarp remained stable throughout fruit development. In other words, JA accumulation was activated in the exocarp, not in the mesocarp, and most particularly only during early stages of fruit development. Similarly, an exocarp-specific SA decrease was observed during fruit development, while this hormone remained at constant levels in the mesocarp. In contrast, ABA contents increased evenly in both tissues. These results highlight the relevance of the fruit exocarp as a more active and dynamic tissue than the mesocarp, where SA and JA shifts are negligible, which could have strong implications in fruit-microbe interactions. On the other hand, it brings forward the issue that hormone analyses on whole fruits, although necessary and informative, should be complemented with tissue specific analyses in order to have a complete and accurate view of hormonal regulation of fruit development. In addition, correlation analyses showed significant corelations between different hormone levels and tissues, suggesting a certain hormone crosstalk between ABA, SA, and JA during fruit development. Indeed, this crosstalk could have not only strong implications in fruit ripening, as previously shown (Fuentes et al., 2019; Tijero et al., 2019), but also in the fruit ability to mount an effective defense against fruit-decay pathogens.

Plant immune responses are characterized by a certain balance between several hormones, including SA, JA, ethylene, and ABA (Robert-Seilaniantz et al., 2011). SA-dependent responses usually confer resistance to biotrophic pathogens, and higher SA levels in the exocarp at early stages of development, as observed in this experiment, could be essential in front of infections by necrotrophic pathogens that remain in the exocarp at their quiescent phase (Prusky et al., 2013). In addition, SA can also confer resistance to necrotrophic pathogens like *Monilinia fructicola* (Yao and Tian, 2005). On the other hand, JA is involved in resistance to nectrotrophic pathogens through the ETHYLENE RESPONSE FACTOR (ERF) branch of JA signaling (Pieterse et al., 2012; Campos et al., 2014), and varying levels of JA during fruit development in the exocarp could affect this response. Finally, ABA has a negative effect on plant resistance. The lack of this hormone increases resistance in tomato plant to *B. cinerea*, and ABA signaling antagonizes with SA-dependent responses and the ERF branch of JA signaling (Asselbergh et al., 2007; Robert-Seilaniantz et al., 2011; Pieterse et al., 2012). Therefore, the tissue-specific variations observed in the exocarp and mesocarp of sweet cherries in our study strongly support the idea that the hormonal changes observed during fruit ripening will influence the susceptibility of fruits to the infection by specific pathogens. Sustained increases in ABA contents both in the exocarp and the mesocarp, together with sustained decreases of SA contents (most particularly in the exocarp) and increases of JA contents in the exocarp (but during early stages of development only), suggest that not only the exocarp, but also the mesocarp, will be most sensitive to pathogen infection during fruit ripening, and contrariwise, it will be better protected from microbial attack during early stages of fruit development. The fact that both JA and SA contents were higher in the exocarp than in the mesocarp also suggests that the exocarp acts not only as a physical barrier but also as an immune barrier against biotrophic and necrotrophic pathogens.

Epiphytic microbial quantification and characterization showed a constant microbial population per area in the sweet cherry skin. However, the epiphytic microbial load per fruit significantly increased during fruit ripening, suggesting an active microbial reproduction on the fruit outer surface. Culturable epiphytic microbial community was mainly composed by yeasts, followed by other fungi and bacteria. Specifically, several fungal decay pathogens were recurrently found from the beginning of fruit development in the epiphytic culturable microbiome, such as *Cladosporium* and *Alternaria* species. These genera have been extensively described as important post-harvest decay pathogens in sweet cherries and other fruits (Granado et al., 2008; Tarbath et al., 2014). Additionally, different *Aureobasidium* isolates were recurrently found. Previous reports have described the biocontrol ability of certain *Aureobasidium* strains against certain post-harvest decay pathogens like *B. cinerea*, but other strains can behave as opportunistic pathogens in apples and sweet cherries (Kim, 2014; Wang et al., 2018). Altogether, our data confirmed that fungal post-harvest decay pathogens were already present at the field not only prior to harvest, but also at very early stages of fruit development.

Some fruit decay fungal genera such as *Botrytis*, *Monilinia*, or *Alternaria* can infect fruits during flowering and at unripe stages of development, behaving as biotrophic pathogens, and remaining in a quiescent state at the fruit outermost layers waiting for the right conditions to become active and cause the disease (Prusky et al., 2013). The early presence of some fruit decay pathogens in sweet cherry development in our study suggests that fungal infections could be taking place as soon as fruits are at stage I of development. Higher SA in the exocarp during early sweet cherry development, as observed in this study, could be an advantage to orchestrate a specific immune response against these infections (Prusky et al., 2013). On the other hand, increased JA levels in the exocarp could be beneficial at later stages of development, when these quiescent infections switch to a necrotrophic lifestyle and as observed in this study, when the microbial epiphytic load substantially increases.

According to our results, endophytic colonization of sweet cherries in the field was not a common event, with few CFU/gFW, suggesting that this colonization could be limited and opportunistic rather than active and widespread. Nevertheless, the onset of fruit ripening could be described as a tipping point in endophytic colonization, as endophytes were only observed in fruits at stages III and at harvest, but not before. Furthermore, endophytic amounts tended to increase throughout development. Several physiological changes take place in sweet cherries during fruit development, and specially at the onset of ripening, including an increase in total soluble sugars, pH decreases and, therefore, an enhanced sugar availability (Prusky et al., 2013; Teribia et al., 2016), which could explain this observation. Our correlation analyses suggest that ABA mesocarp content could be an additional key factor in regulating this endophytic colonization, although its exact molecular mechanism could not yet be unraveled. It is likely that the increase of ABA during ripening may have an antagonistic effect in front of SA and JA-dependent immune responses, limiting the fruit to mount an effective defense response (Asselbergh et al., 2007; Pieterse et al., 2012; Alkan and Fortes, 2015), but further investigations will be required to support this contention.

Fruit cracking is characterized by a deep disruption of the fruit exocarp (Correia et al., 2018). A microbiological analysis of cracked cherries revealed a heavy colonization by the rot-causing fungi *Alternaria* spp., and other microorganisms like *Aureobasidium* spp. Interestingly, these microorganisms were present as epiphytes on the fruit skin during fruit development, but never as endophytes in healthy cherries. This proves that the exocarp is a physical barrier not only for pathogenic microorganisms, but also for a wide variety of microbes, and when it is broken, it can lead to fruit colonization by different pathogenic and non-pathogenic microorganisms. Several studies have aimed to prevent sweet cherry cracking to reduce fruit loss in the field (Thomidis and Exadaktylou, 2013; Correia et al., 2018). Recently, it was reported that exogenous application of ABA at early stages of development could reduce sweet cherry cracking (Balbontín et al., 2018). Paradoxically, even though ABA could have negative effects on plant immune responses to pathogens, it could indirectly be beneficial by preventing fruit cracking and subsequent fruit infection by pathogens.

In conclusion, tissue-specific hormone variations may be very useful to better understand fruit-microbiome interactions, which may have important implications to reduce yield losses in sweet cherry commercial orchards. Our results suggest a differential SA and JA hormone behavior between tissues, suggesting that fruit exocarp is a key tissue in fruit development and in fruit-microbe relations, not only by acting as a physical barrier in front of epiphytic pathogens, but also providing important hormonal signals that influence fruit-microbiome interactions. Further research is, however, needed to better understand putative causal relationships between specific hormonal variations in the exocarp and mesocarp and the increased occurrence of pathogen infection during fruit ripening. A better exploration of possible microbiome interactions is also required to better understand possible biocontrol strategies to prevent fruit decay in sweet cherries.

## Data Availability Statement

The raw data supporting the conclusions of this article will be made available by the authors, without undue reservation.

## Author Contributions

DF and SM-B conceived and designed the experiments. DF performed the experiments and wrote the manuscript with the help of SM-B. Both authors contributed to the discussion and revised and approved the final manuscript.

### Conflict of Interest

The authors declare that the research was conducted in the absence of any commercial or financial relationships that could be construed as a potential conflict of interest.
