# Supplementary material for: Differential Tissue-Specific Jasmonic Acid, Salicylic Acid, and Abscisic Acid Dynamics in Sweet Cherry Development and Their Implications in Fruit-Microbe Interactions
Source: Front Plant Sci. 2021 Feb 2;12:640601. doi: 10.3389/fpls.2021.640601 (PMC7884454; doi:10.3389/fpls.2021.640601)
Supplement: Supplementary file 1 [file Data_Sheet_1.pdf]

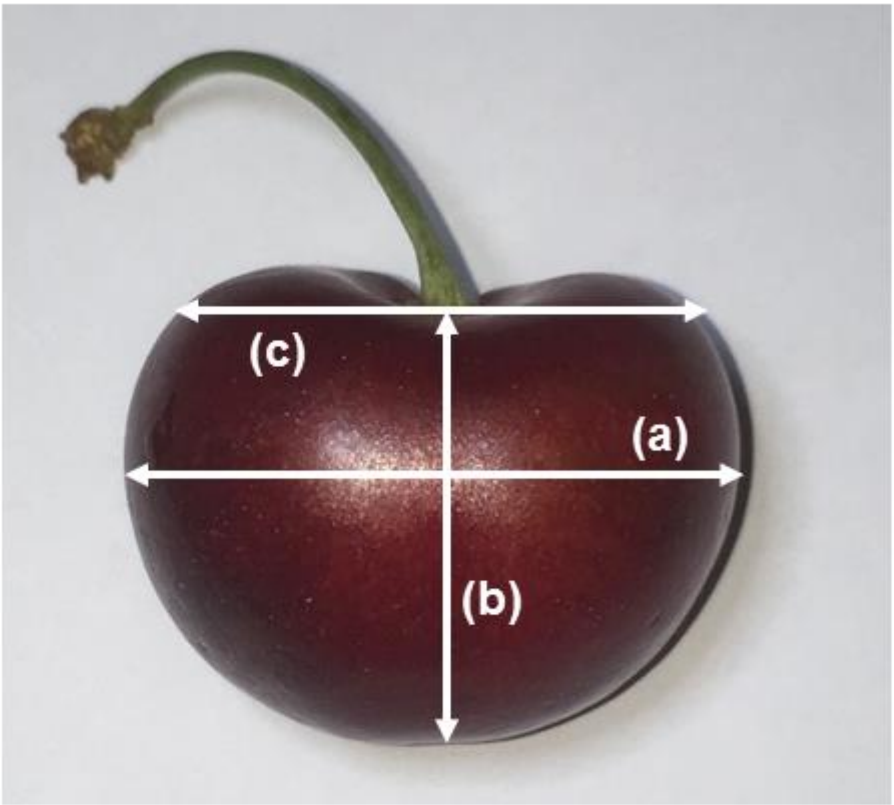

Suppl. Fig. 1. Fruit width (a), height from the apex to the base of the peduncle (b), and width at the top of the fruit (c) were used to estimate total fruit skin surface.

Suppl. Table 1

Morphological characterization of the most frequent epiphytic and endophytic microbial isolates from the exocarp and mesocarp of commercial orchard sweet cherry fruits. Microbial isolates from cracked cherries are also indicated.

Isolate	Tissue source	Microbial group	Colony characteristic				
			Shape	Margin	Colour	Consistency	Other
Ep-1	Exocarp	Yeast	Round	Entire	Cream-Pink	Butyrous	
Ep-2	Exocarp<br>Mesocarp[a]	Yeast	Round	Entire	Pink-Red	Mucoid	
Ep-4	Exocarp	Bacteria	Irregular	Undulate	Yellow	Mucoid	Bacterial morphology: Rods
En-1	Mesocarp	Yeast	Round	Entire	White-Cream Pink	Butyrous	
En-2	Mesocarp[a]	Yeast	Irregular	Entire	Off White-Yellow	Butyrous	

Note: morphological characterization was performed according to Krishnan et al. 2012.

[a] Isolates were found in the mesocarp of cracked cherries, but not in the mesocarp of healthy ones.
